# Validation of a food frequency questionnaire for estimating vitamin K intake in the overweight adult Mexican population

**DOI:** 10.1186/s40795-025-01187-y

**Published:** 2025-11-03

**Authors:** Xochitl Citlalli Olivares-Ochoa, Fabiola Márquez-Sandoval, Edgar Alfonso Rivera-León, Erika Martínez-López, Andres López-Quintero, Yahatziri Salinas-Varela, Iris Monserrat Llamas-Covarrubias

**Affiliations:** 1 Doctorado en Ciencias de la Nutrición Traslacional, Centro Universitario de Ciencias de la Salud, University of Guadalajara, Guadalajara, México; 2 Instituto de Nutrigenética y Nutrigenómica Traslacional, Centro Universitario de Ciencias de la Salud, University of Guadalajara, Guadalajara, México; 3 División de Ciencias Biomédicas, Centro Universitario de Los Altos, University of Guadalajara, Tepatitlán de Morelos, Tepatitlán, México; 4 Departamento de Biología Molecular y Genómica, Centro Universitario de Ciencias de la Salud, University of Guadalajara, Guadalajara, México

**Keywords:** Food frequency questionnaire, Vitamin K, Mexican adults

## Abstract

**Background:**

It is important to assess vitamin K intake due to its involvement in multiple physiological processes. This study aims to validate a food frequency questionnaire for estimating vitamin K1, vitamin K2 and total vitamin K intake over 3 months in the adult Mexican population.

**Methods:**

The food frequency questionnaire design included 49 food items and six frequency categories. The study included 42 young adults from Mexico classified as overweight or obese. Participants completed the food frequency questionnaire twice over a three-month period (at baseline and at the end of the study), and reproducibility was assessed using these measurements. Additionally, four 24-hour dietary recalls were collected throughout the same period to evaluate criterion validity.

**Results:**

The final food frequency questionnaire showed good relative and absolute agreement in estimating vitamin K1 intake compared to the average of four 24-hour dietary recalls. In the estimation of vitamin K2 and total vitamin K intake, discrepancies in the agreement between the tools were observed.

**Conclusions:**

This study presents the first approach to developing a food frequency questionnaire for estimating vitamin K intake in the adult Mexican population. Its use is recommended for estimating vitamin K1 intake in both clinical practice and research. It is crucial to obtain more comprehensive and accurate information on food composition, both in Mexico and globally, to more precisely assess vitamin K2 intake in different populations.

**Supplementary Information:**

The online version contains supplementary material available at 10.1186/s40795-025-01187-y.

## Background

Vitamin K (VK) refers to a group of fat-soluble organic compounds. It exists in two biologically active natural forms: vitamin K1 (VK1) and vitamin K2 (VK2) [1]. These isoforms share structural similarities, including a bicyclic core and a variable side chain at position 3 of the quinone ring [2]. VK1, or phylloquinone, is primarily found in green leafy vegetables and has a branched aliphatic side chain with a double bond. VK2 includes a variety of forms collectively known as menaquinones. It is produced through bacterial synthesis, meaning the human body can also produce it. This isoform is found in fermented foods and animal products such as meat and dairy. VK2 has an isoprenoid side chain with varying numbers of carbon atoms, which determine its name, ranging from menaquinone-2 to menaquinone-15 [2–4].

VK is essential in various physiological processes within the human body, as it serves as an enzymatic cofactor for the gamma-carboxylation of VK-dependent proteins [5]. Its role in haemostasis is well known, but it also plays an important part in numerous biochemical and biological functions such as inflammation, oxidation, bone metabolism, and tissue mineralization, vital functions for maintaining health [3]. The relationship between VK1 and the coagulation process is well-established. Its half-life is up to 24 h, which promotes its storage and function within liver tissue. In contrast, VK2 has a longer half-life (up to 72 h). This feature allows it to remain in the bloodstream longer and act on extrahepatic tissues, such as bones and the vascular system, which links it to other effects of VK, such as bone metabolism and tissue mineralization [6].

The adequate intake, according to the Institute of Medicine (IOM), is 90 µg/day for adult women and 120 µg/day for adult men [7]. This recommended intake is based on the requirements of VK1 for the functioning of coagulation factors, so the amount needed for other processes may be higher [8]. Both isoforms contribute to the body VK status, and while VK1 is considered the main contributor in the Western diet, its absorption is lower compared to VK2 [3].

There are tools designed to estimate nutrient intake, including the semi-quantitative food frequency questionnaire (FFQ), which, due to its ease of use and ability to assess intake over a period of time, is the most commonly used [9, 10]. These questionnaires have been used to study the relationship between VK and cardiovascular diseases, bone disorders, and cancer. However, these studies use FFQs designed to estimate overall dietary intake, which may lead to a loss of information when assessing a single nutrient like VK, which is found in a limited number of food sources [11].

FFQs have been designed and validated to estimate VK intake in various populations [12–18]. Nevertheless, applying these FFQs to the Mexican population would not be ideal, as the FFQ should be tailored to the target population, since eating habits vary by region depending on food availability and cultural practices [11]. Furthermore, only two of these FFQs include the estimation of VK2 intake [12, 18]. An FFQ has been validated in the Mexican population to estimate the intake of food groups and nutrients [19]. The limitation of this tool is that it does not include the estimation of certain nutrients, such as VK. Therefore, this study aims to validate an FFQ for estimating VK1, VK2 and total VK intake over a 3-month period in the adult Mexican population.

## Methods

The methodology applied in this research aligns with the COSMIN (Consensus-based Standards for the Selection of Health Measurement Instruments) guidelines [20]. These standards provide a structured framework for evaluating validity and reliability in health-related measurement tools. Specifically, we assessed content and construct validity.

### Design of the FFQ

**Fig. 1:**
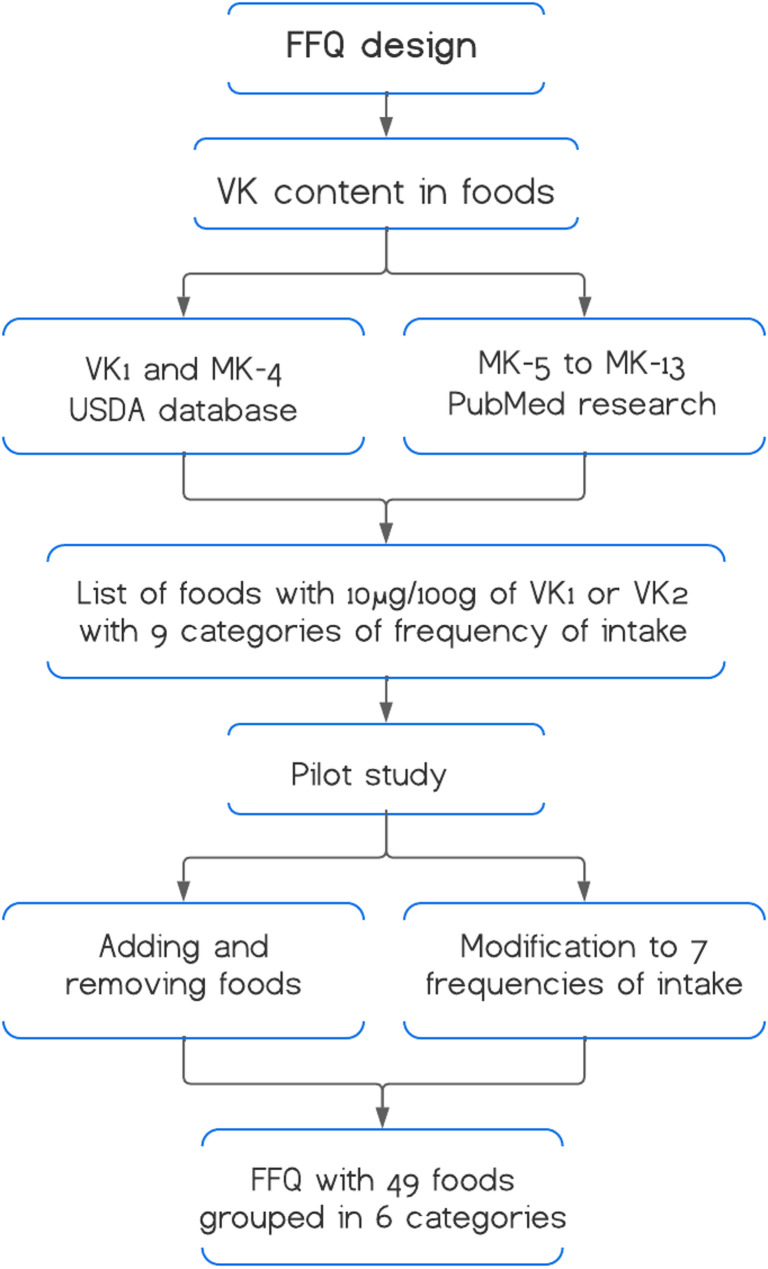
Design of the FFQ

A review of the available literature was conducted to determine the VK content in foods. The VK1 and menaquinone-4 content in the FFQ was based on values reported in the 2019–2020 Food and Nutrient Database for Dietary Studies from the United States Department of Agriculture (USDA) [21]. Since this database does not report the content of other menaquinones, a search was conducted in the PubMed database for menaquinones 5–13. The keywords ‘Vitamin K,’ ‘content,’ and ‘food’ were used, yielding 336 results. After screening the articles, 7 studies published in the last 10 years were selected, which determined the content of these nutrients (Fig. 1) [22–28].

The foods included in the FFQ were selected based on the identification of foods rich in VK1 and VK2 available in the region. The minimum content required for inclusion was 10 µg/100 g food of VK1 or VK2. The frequency of food consumption was initially grouped into nine categories based on the FFQ for the Mexican population, as proposed by Willett: ‘never or almost never,’ ‘1–3 times per month,’ ‘once a week,’ ‘2–4 times a week,’ ‘5–6 times a week,’ ‘1 serving per day,’ ‘2–3 servings per day,’ ‘4–6 servings per day,’ and ‘more than six servings per day’ [29].

To construct the questionnaire, three available validated tools were considered: a semi-quantitative FFQ used in the Mexican population to assess food groups and nutrient intake, and two semi-quantitative FFQs used in the U.S. adults and Mediterranean populations to assess daily VK intake [12, 14, 19]. The three FFQs mentioned were reviewed to understand the methodological foundations for designing our questionnaire. They served as a reference to identify which foods could be considered, typical frequency categories, and general structure.

The comprehension and relevance of the initial FFQ were evaluated through a pilot test conducted with 20 individuals with similar characteristics to those in the main study. The questionnaire was administered alongside a 24-hour dietary recall (24HDR), using the same methodology described in the reference method section. After evaluating the results, some foods not included in the initial FFQ were identified (chayote, apple, tomato, plum, pork, ham, milk, and eggs). Although these foods did not contain a specified amount of VK, they were added to the tool because they were frequently consumed according to the 24HDR. Other foods (brussels sprouts and dried figs) were excluded due to the absence of consumption among participants. After review by experts and following their suggestions, the frequency categories were changed to seven categories to better reflect the temporal aspect. The selected categories were: ‘never or almost never,’ ‘1–2 times per quarter,’ ‘1–2 times per month,’ ‘once a week,’ ‘2–4 times per week,’ ‘5–6 times per week,’ and ‘daily’.

Based on the changes from the previous process, an FFQ was redesigned as a dietary tool to estimate daily intake of VK1, VK2, and total VK over a 3-month period. The tool includes 49 foods grouped into vegetables, fruits, legumes, animal-based foods, and fats and oils, both with and without protein (Additional file 1).

### Reference method

The 24HDR was used as the reference method during this validation. This tool was applied using the same methodology as the FFQ, where the administrator explained the purpose of the tool and emphasized the recording of all foods consumed on the previous day. All foods consumed were recorded by mealtimes, and later the quantity, type, and brand were noted. Portion sizes were estimated using standardized household measure replicas (e.g., cups, spoons, and bowls of different sizes), which were shown to participants during the interviews to help them more accurately report the amounts of foods and beverages consumed.

### Data collection

For the validation process the selected participants came from a previous study registered on ClinicalTrials.gov with the identifier NCT05995522. Eligible volunteers were young adults with a body mass index (BMI) between 25 and 40 kg/m² but otherwise apparently healthy. A total of 42 adults were selected through a convenience sampling method.

Data collection took place at a university. A healthcare professional administered the FFQ electronically, which took approximately 10 minutes. Before starting the FFQ, participants were given an introduction explaining its purpose and emphasising that their responses should reflect their food consumption frequency over the past 3 months. Once the participant indicated the frequency, the amount consumed per occasion was recorded. The validated ‘Mexican Food Photographic Album’ was used as a visual aid, following its established methodology [30]. Some foods included in the FFQ were not found in the album, so additional photographs were taken using the same methodology to standardise the process.

A database was created in Excel (Microsoft 365 MSO, Version 2407, Redmond, WA, USA) with VK1 and VK2 content in foods. The intake of menaquinones was summed and reported as VK2, while total VK intake was the sum of VK1 and VK2. Daily intake (g/day) was calculated using the formula: nutrient content per serving × serving size × frequency conversion factor (Additional file 2).

**Fig. 2:**
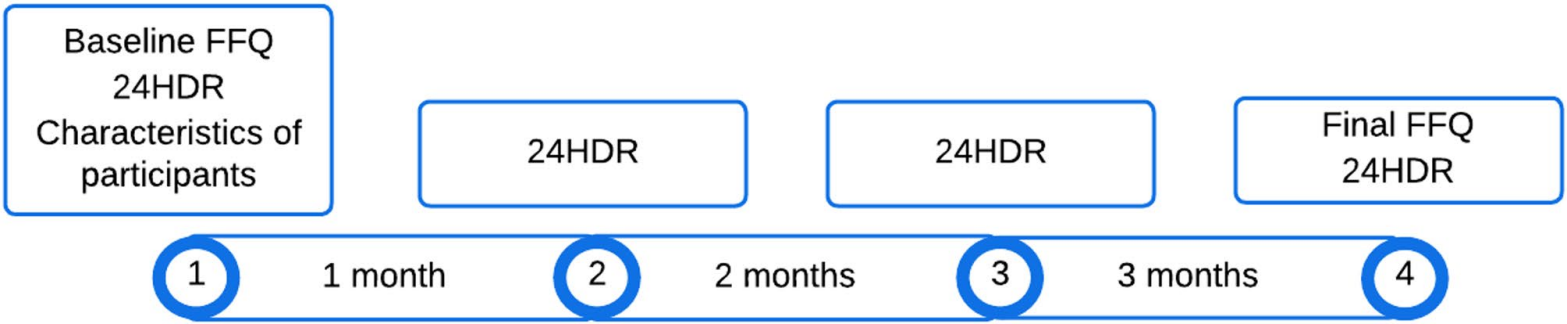
Timing of tool applications

The FFQ was administered for the first time (baseline FFQ) during the initial interview and again after 3 months, following the same methodology (final FFQ) (Fig. 2). In parallel with the FFQ, 24HDRs were recorded throughout the 3-month follow-up period.

Different variables were assessed in the participants to define the characteristics of the study population. Below are the details:

### Sociodemographic and anthropometric variables

We assessed education status, which was categorised as completed high school, bachelor’s degree, and postgraduate (including master’s and doctoral degrees). Anthropometric variables were also measured, including weight and height to calculate BMI using the formula: weight (kg)/height² (m). Weight was measured using a Tanita TBF-300 Scale (Tanita Corporation, Arlington Heights, IL, USA), and height was measured with a portable mechanical stadiometer Seca Model 213 (Seca GmbH & Co. KG, Hamburg, Germany). Waist circumference was measured at the midpoint between the lower rib and the upper edge of the iliac crest along the mid-axillary line, using a Lufkin Metal Tape Measure (Lufkin, Grand Prairie, TX, USA).

### Biochemical variables

Blood samples were collected in the morning after an eight-hour fast. Serum levels of total cholesterol, HDL cholesterol, triglycerides, and glucose were analyzed using the VITROS^®^ 350 System (Ortho Clinical Diagnostics, Raritan, NJ, USA). The LDL cholesterol value was calculated by the system using Friedewald’s formula: LDL cholesterol = total cholesterol - HDL cholesterol - (triglycerides/5). Serum insulin levels were determined by enzyme-linked immunosorbent assay with insulin ELISA (DRG International, Inc., Springfield, NJ, USA).

### Statistical analysis

Normality of the variables was assessed using the Shapiro-Wilk test. Variables that were not normally distributed were log-transformed for statistical analysis and subsequently retransformed for result presentation. Qualitative variables were expressed as frequencies and percentages, while quantitative variables were expressed as means and standard deviations or medians and ranges. Comparisons between groups (men vs. women) were performed using the independent t-test and Fisher’s exact test. Differences between tools were analysed using the independent t-test to estimate criterion validity. Additionally, Bland-Altman plots, Pearson’s correlation test, and the intraclass correlation coefficient (reproducibility) were calculated. Statistical analysis was conducted using SPSS^®^ Statistics 25.0 (IBM Corp., Armonk, NY, USA) for Windows, with statistical significance set at *p* < 0.05.

## Results

This study included 42 healthy young adults, as self-reported. The mean age was 26.92 ± 4.02 years, with 54.8% of the participants being male (Table 1). Regarding education level, most participants held a bachelor’s degree (66.7%). 26% of the men had postgraduate education, compared to 0% of the women; however, no significant differences were found between sexes in this variable. Participants had a mean BMI of 30.76 ± 3.81 kg/m². Statistically, women had lower values for waist circumference and lipid profiles compared to men.

**Table 1:** Characteristics of the participants

Variable	All (*n* = 42) Mean ± SD	Males (*n* = 23) Mean ± SD	Females (*n* = 19) Mean ± SD	*p*
Sociodemographic variables				
Age (years)	26.92 ± 4.02	27.86 ± 3.57	25.78 ± 4.35	0.097^a^
Education n (%)				0.051^b^
High school	8 (19.0)	4 (17.4)	4 (21.1)	
Bacherol’s degree	28 (66.7)	13 (56.5)	15 (78.9)	
Postgraduate	6 (14.3)	6 (26.1)	0 (0)	
Anthropometric variables				
BMI (kg/m^2^)	30.76 ± 3.81	30.25 ± 3.57	31.37 ± 4.08	0.347^a^
Waist circumference (cm)	95.50 ± 10.11	98.71 ± 8.57	91.62 ± 10.68	**0.022**^**a**^
Biochemical variables				
Glucose (mg/dL)	88.28 ± 9.73	88.86 ± 10.39	87.57 ± 9.11	0.674^a^
Insulin (uIU/mL)^c^	20.50 ± 11.52	20.72 ± 11.94	20.24 ± 11.30	0.970^a^
Triglycerides (mg/dL)^c^	156.09 ± 85.40	194.73 ± 92.83	109.31 ± 43.14	**< 0.001**^**a**^
Total cholesterol (mg/dL)	171.38 ± 36.70	188.47 ± 37.16	150.68 ± 23.54	**< 0.001**^**a**^
HDL cholesterol (mg/dL)	38.35 ± 10.06	36.04 ± 10.36	41.15 ± 9.19	0.102^a^
LDL cholesterol (mg/dL)	101.80 ± 30.35	113.52 ± 33.20	87.63 ± 19.07	**0.003**^**a**^

*BMI *Body mass index. Data comparisons: Result obtained by (a) Independent samples t-test, (b) Fisher’s exact test. (c) Variables log-transformed for normality and then re-transformed

Regarding baseline VK intake, the total intake of VK, estimated using the baseline FFQ, was 151.56 ± 129.76 µg/day, while according to the baseline 24HDR, it was 114.32 ± 109.69 µg/day (Additional file 3). The FFQ estimated a significantly higher intake of VK1, VK2, and total VK compared to the 24HDR.

In terms of validity, when comparing the final FFQ with the average of the four 24HDR, the final FFQ reported a higher average intake for all VK isoforms (Table 2). The estimated VK1 intake was 10.80 ± 79.14 µg/day higher than the final FFQ compared to the 24HDR. However, this difference was not statistically significant.

**Table 2:** Comparison of VK intake according to the average of 4 24HDR and final FFQ

VK isoform (µg/day)	4-day averaged 24HDR (*n* = 42) Mean ± SD Median [25th-75th]	Final FFQ (*n* = 42) Mean ± SD Median [25th-75th]	*p*^a^	*r*	*p*^b^
VK1	95.02 ± 59.31 81.32 [47.07–123.25.07.25]	105.83 ± 65.71 82.04 [53.99–150.06.99.06]	0.430	**0.327**	**0.035**
VK2	49.50 ± 34.02 39.81 [26.14–65.75]	76.25 ± 38.87 68.87 [45.40–103.54.40.54]	**0.001**	0.011	0.947
Total VK	144.53 ± 62.37 135.42 [96.55–181.25.55.25]	181.68 ± 74.47 177.61 [114.33–234.75.33.75]	**0.016**	0.163	0.304

*24HDR *24-hour dietary recall, *FFQ *Food frequency questionnaire, *VK1 *Vitamin K1, *VK2 *Vitamin K2, *VK *Vitamin K. Data comparisons: Result obtained by (a) Independent samples t-test, (b) Pearson correlation. Variables log-transformed for normality and then re-transformed

**Fig. 3:**
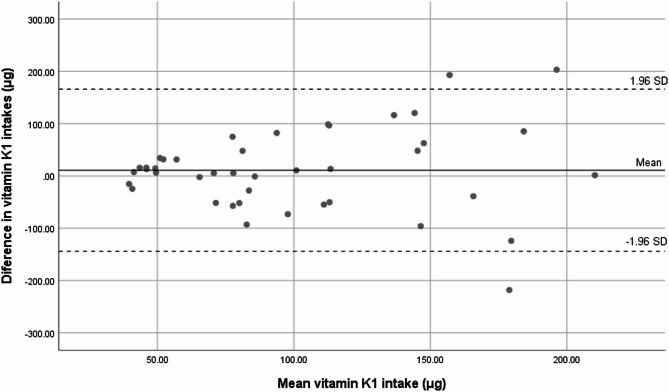
Agreement in VK1 intake between the 4-day averaged 24HDR and the final FFQ

A statistically significant correlation was observed between the two methods (*r* = 0.327; *p* = 0.035). At the individual level, the Bland-Altman plot showed that most participants were within the limits of agreement, with two outliers above the upper limit and one participant outside the lower limit (Fig. 3).

Regarding the reproducibility of VK1 intake, we observed a significant (*p* = 0.014) but low correlation between baseline and final FFQ with an intraclass correlation coefficient (ICC) of 0.337 (95% CI = 0.040; 0.579) (Table 3).

**Table 3:** Reproducibility at 3 months for VK intake based on the baseline and final FFQ

VK isoform (µg/day)	Baseline FFQ (*n* = 42) Mean ± SD Median [25th-75th]	Final FFQ (*n* = 42) Mean ± SD Median [25th-75th]	CCI	IC 95%	*p*
VK1	151.56 ± 129.76 110.26 [68.75–195.35.75.35]	105.83 ± 65.71 82.04 [53.99–150.06.99.06]	0.337	0.040; 0.579	**0.014**
VK2	82.53 ± 49.96 76.10 [45.19–111.09.19.09]	76.25 ± 38.87 68.87 [45.40–103.54.40.54]	0.515	0.253; 0.706	**< 0.001**
Total VK	233.12 ± 133.60 195.66 [130.92–331.74.92.74]	181.68 ± 74.47 177.61 [114.33–234.75.33.75]	0.375	0.154; 0.756	**0.007**

*24HDR *24-hour dietary recall, *FFQ *Food frequency questionnaire, *CCI *Intraclass correlation coefficient. IC 95%: 95% confidence interval; *VK1 *Vitamin K1, *VK2 *Vitamin K2, *VK *Vitamin K. The result was obtained using an ICC

The final FFQ estimated a significantly higher intake of VK2 (26.74 ± 50.84 µg/day) and total VK (37.15 ± 93.67 µg/day) compared to the average of the 24HDR. The results showed a low correlation between the two methods. On the other hand, the Bland-Altman plot indicated that most participants were within limits of agreement (Additional file 4). Significant moderate correlations between the baseline and final FFQ for VK2 and total VK were observed in the reproducibility.

## Discussion

In the field of human nutrition, assessing VK intake is crucial due to its involvement in various physiological processes [3, 6]. The FFQ is the most used tool for estimating nutrient intake because of its many advantages [10, 12]. The FFQ designed in this study was found to be valid for estimating VK1 intake when compared to the average of the four 24HDR. Regarding relative concordance, a significant correlation coefficient of 0.32 was observed, surpassing the minimum threshold of 0.3 recommended for FFQ validation studies [31]. Previous studies assessing VK1 intake in adult populations from Canada, Brazil, and the Netherlands report correlation coefficients ranging from 0.2 to 0.8 [13, 16–18].

In VK2 intake estimation, the FFQ showed discrepancies in both relative and absolute agreement when compared to the average of the four 24HDR. The correlation coefficient for VK2 intake was low (*r* = 0.011, *p* = 0.947). Only one validated FFQ in the Dutch adult population has assessed VK2 intake, reporting a correlation coefficient of 0.39 for women and 0.52 for men [18]. Like the findings for VK2, the total VK intake estimated by the FFQ showed low relative (*r* = 0.163, *p* = 0.304) and absolute concordance with the estimate from the average of the four 24HDR. Two studies conducted in adult populations in the Netherlands and Portugal evaluated total VK intake, reporting correlation coefficients of 0.24, 0.46, and 0.69 [12, 18].

The discrepancy between the previous results could be attributed to the fact that while VK1 content in foods has been extensively studied, and there are food composition databases with determinations of this isoform, VK2 content is less well-documented, limiting the ability to estimate its intake [32]. In contrast to the study by Zwakenberg et al. [18], which analysed regional data on VK2 content in foods, this study relied on data from other countries for the analysis of VK2 intake due to the lack of data on Mexican foods. In addition to the fact that VK2 content in foods is influenced by geographic location, there may be Mexican foods with significant VK2 content that were not included in the FFQ due to the lack of available information [28]. The results observed for total VK intake were as expected, since total VK intake includes VK2 intake, and the findings were consistent with this.

In the reproducibility of the FFQ, we found an ICC of 0.33 for VK1, indicating poor reliability [33]. Only one study conducted in the Netherlands has evaluated reproducibility using a test-retest approach with the ICC, reporting a coefficient of 0.70 in men and 0.65 in women [18]. The difference in results may be since our FFQ was evaluated in 3 months, whereas the previous study assessed it at 12 months. This is important because, although it has been suggested that VK1 intake does not vary substantially throughout the year, studies report high variability within individuals in both VK1 and green vegetable intake [34–36]. VK2 showed an ICC of 0.51, categorised as moderate reliability. The FFQ validated in the Dutch population reported a coefficient of 0.73 in men and 0.79 in women, with a similar interpretation to that found in this study [18].

There is limited data on VK intake in the Mexican population. According to the final FFQ, the average VK1 intake was 106.29 ± 65.00 µg/day, with 65.1% of participants consuming less than the adequate intake level established by the IOM [7]. This is similar, a previous study involving young Mexican adults reported that 58.2% of participants did not meet the adequate intake, based on the same criteria [37]. Higher VK intakes have been documented in other populations, with reported averages ranging from 112.55 ± 82.66 µg/day up to 287 ± 228 µg/day among adults in the Netherlands, USA, Brazil, Korea, and Canada [13–18].

The methods used in this study have been widely recommended and adopted for FFQ validation. The 24HDR, as the reference method, is highly regarded because it provides detailed dietary information [9]. Additionally, the use of tools with photographic support as a visual aid is a technique that has been successfully employed by several researchers [12, 15, 18]. Furthermore, the analysis of relative and absolute concordance between the FFQ and reference methods has been a standard approach in numerous FFQ validations, both for VK and other nutrients [12, 16, 18, 38, 39].

Some limitations were identified in this study that could be addressed in future research on the topic. The FFQ has difficulties estimating VK2 intake, possibly due to the lack of data on the content of this isoform in foods. It is crucial to obtain more comprehensive and accurate information on food composition, both in Mexico and globally, to more precisely assess VK2 intake in different populations. In the meantime, the results obtained regarding VK2 intake should be interpreted with caution [32].

In this validation, the use of four 24HDR as the reference method was considered. However, evidence suggests using at least five applications at different times to improve the accuracy of habitual VK intake [13]. Other FFQ validations for VK have used between 2 and 12 applications, with 5 being the most common number of repetitions [12–18].

We performed a sample size calculation following the methods proposed by Hulley et al. [40], resulting in a minimum sample size of 17 individuals. A statistical power of 90%, a minimum correlation coefficient of 0.697 previously reported between total vitamin K intake estimated by the FFQ and the reference method [12], and a significance level of 5% were considered. Since additional participants were available from another study, we opted to use convenience sampling. Convenience sampling is a non-probabilistic technique commonly used in research due to its ease of access to participants. In this case, young Mexican adults with overweight or obesity were selected. Therefore, the findings may not be generalizable to other populations.

This study represents a valuable starting point for the development and refinement of future tools, as the use of instruments without proper validation carries significant risk due to the lack of knowledge about their limitations, which can lead to biases in dietary intake measurement. Initiatives like this help disseminate the measurement properties of food frequency questionnaires, facilitating access to instruments with known validity data.

While reproducibility has room for improvement, it is important to highlight that most validation studies of FFQs for vitamin K do not assess temporal stability, unlike other food frequency questionnaires designed to evaluate general dietary intake, where this aspect has been more commonly addressed [41]. Therefore, including it in this study provides valuable insight into the questionnaire’s consistency over time.

Building on these findings and aiming to further improve this tool, we recommend the continued application and validation of this FFQ in diverse populations and with varying sample sizes. Repeated administrations of the reference method and more precise estimates of vitamin K2 content in commonly consumed Mexican foods are also necessary to enhance its reproducibility and validity. Future research should prioritize evaluating and refining the questionnaire across broader demographic groups, including different geographic regions, age ranges, and nutritional statuses. As with all instruments, expanding validation efforts will support the continuous improvement of the tool and increase its relevance and applicability to other populations.

To the best of our knowledge, this is the first validated FFQ for VK in the Mexican population, representing a significant contribution to the scientific literature due to the lack of prior studies in this specific context. The validation of this FFQ in a local population allows for more accurate estimates of VK intake in the Mexican population. Furthermore, the authors of the study emphasise the relevance of analysing VK2 intake, which has traditionally been less studied compared to VK1. By including this isoform in their analysis, the study expands knowledge on the consumption patterns of both VK forms, which is essential for future research and the improvement of dietary recommendations.

The applications of this FFQ are numerous, both in clinical practice and in research. VK1 is closely related to coagulation and is particularly relevant to its intake in patients undergoing anticoagulant therapy [6]. Warfarin, a medication that acts as a VK antagonist, interferes with its metabolism, making its intake a determining factor in the effectiveness of the treatment [42]. Additionally, various FFQs designed to estimate VK1 intake have been used in the prevention and management of diseases such as osteoporosis and cardiovascular diseases, or simply to assess VK intake over a specific period [15, 43].

## Conclusions

This study presents the first approach to developing a food frequency questionnaire for estimating vitamin K intake in the adult Mexican population. Its use is recommended for estimating VK1 intake in both clinical practice and research.

## Supplementary Information

Supplementary Material 1.

Supplementary Material 2.

Supplementary Material 3.

Supplementary Material 4.

## Data Availability

Raw data supporting the findings of this study are available upon reasonable request from the corresponding author to ensure participant anonymity and comply with data protection regulations.

## Abbreviations

VK: Vitamin K
VK1: Vitamin K1
VK2: Vitamin K2
IOM: Institute of Medicine
FFQ: Food frequency questionnaire
COSMIN: Consensus-based Standards for the Selection of Health Measurement Instruments
USDA: United States Department of Agriculture
24HDR: 24-hour dietary recall
BMI: Body mass index
ICC: Intraclass correlation coefficient
