# Supplementary material for: Validation of a food frequency questionnaire for estimating vitamin K intake in the overweight adult Mexican population
Source: BMC Nutr. 2025 Nov 3;11:201. doi: 10.1186/s40795-025-01187-y (PMC12581294; doi:10.1186/s40795-025-01187-y)
Supplement: Supplementary file 1 — Supplementary Material 1. [file 40795_2025_1187_MOESM1_ESM.pdf]

Frequency conversion factor

Frequency of Intake	Conversion Factors
Never or almost never	0
1-2 times per quarter	0.02
1-3 times per month	0.07
Once a week	0.14
2-4 times per week	0.43
5-6 times per week	0.79
Once a day	1
