# Supplementary material for: Validation of a food frequency questionnaire for estimating vitamin K intake in the overweight adult Mexican population
Source: BMC Nutr. 2025 Nov 3;11:201. doi: 10.1186/s40795-025-01187-y (PMC12581294; doi:10.1186/s40795-025-01187-y)
Supplement: Supplementary file 2 — Supplementary Material 2. [file 40795_2025_1187_MOESM2_ESM.pdf]

Dietary sources of VK included in the FFQ

Food group	Food	VK1 content (μg/100 g)	VK2 content (μg/100 g)
Vegetables	Swiss chard	830	0
	Arugula	108.6	0
	Watercress	541.9	0
	Broccoli	105.9	0
	Chayote	4.1	0
	Peas	25.8	0
	Cabbage	76	0
	Cauliflower	16.1	0
	Asparagus	44.6	0
	Spinach	482.9	0
	Tomato	7.5	0
	Kale	390	0
	Lettuce	75.2	0
	Cucumber	16.4	0
	Parsley	1640	0
	Tomato	8.4	0
	Carrot	13.2	0
Fruits	Dried cranberry	59.4	0
	Plum	6.4	0
	Prune	59.5	0
	Kiwi	40.3	0
	Apple	2.2	0
	Blackberries	19.3	0
	Grapes	14.6	0
Legumes	Beans	10.3	0
	Chickpeas	10.8	0

	Soybeans	25	0
Animal-based foods	Beef meat	1.5	18.9
	Pork meat	0	3.7
	Chicken meat	0	27
	Ham	0	1.4
	Salami	1.3	16
	Beef liver	3.9	112.4
	Gouda cheese	2.3	48.9
	Cottage cheese	0	51.9
	Cheddar cheese	2.4	278.6
	Roquefort cheese	2	380.7
	Regular yogurt	0.2	23.9
	Cow's milk	0.3	1
	Egg	5	0.2
Protein-free fats and oils	Olive oil	26	0
	Canola oil	71.3	0
	Sunflower oil	5.4	0
	Avocado	21	0
	Heavy cream	1.3	583.4
	Margarine	99.2	9.9
	Mayonnaise	163	0
	Bacon	2.8	16
Protein-containing fats and oils	Pistachios	13.2	0
