# Supplementary material for: Validation of a food frequency questionnaire for estimating vitamin K intake in the overweight adult Mexican population
Source: BMC Nutr. 2025 Nov 3;11:201. doi: 10.1186/s40795-025-01187-y (PMC12581294; doi:10.1186/s40795-025-01187-y)
Supplement: Supplementary file 3 — Supplementary Material 3. [file 40795_2025_1187_MOESM3_ESM.pdf]

Comparison of VK intake according to the baseline 24HDR and FFQ

**VK isoform (µg/day)**	**Baseline 24HDR (n=42)**<br>**Mean ± SD**<br>**Median [25th-75th]**	**Baseline FFQ (n=42)**<br>**Mean ± SD**<br>**Median [25th-75th]**	**p[a]**	**r**	**p[b]**
VK1	114.32 ± 109.69<br>76.78 [38.76-168.62]	151.56 ± 129.76<br>110.26 [68.75-195.35]	**0.032**	**0.439**	**0.004**
VK2	34.73 ± 50.84<br>10.68 [2.51-46.78]	82.53 ± 49.96<br>76.10 [45.19-111.09]	**<0.001**	0.301	0.052
Total VK	149.05 ± 119.28<br>117.89 [48.37-199.30]	233.12 ± 133.60<br>195.66 [130.92-331.74]	**<0.001**	0.276	0.077

Abbreviations: 24HDR: 24-hour dietary recall; FFQ: Food frequency questionnaire; VK1: Vitamin K1; VK2: Vitamin K2; VK: Vitamin K. Data comparisons: Result obtained by a) Independent samples t-test, b) Pearson correlation. Variables log-transformed for normality and then re-transformed.
