# Supplementary material for: Validation of a food frequency questionnaire for estimating vitamin K intake in the overweight adult Mexican population
Source: BMC Nutr. 2025 Nov 3;11:201. doi: 10.1186/s40795-025-01187-y (PMC12581294; doi:10.1186/s40795-025-01187-y)
Supplement: Supplementary file 4 — Supplementary Material 4. [file 40795_2025_1187_MOESM4_ESM.pdf]

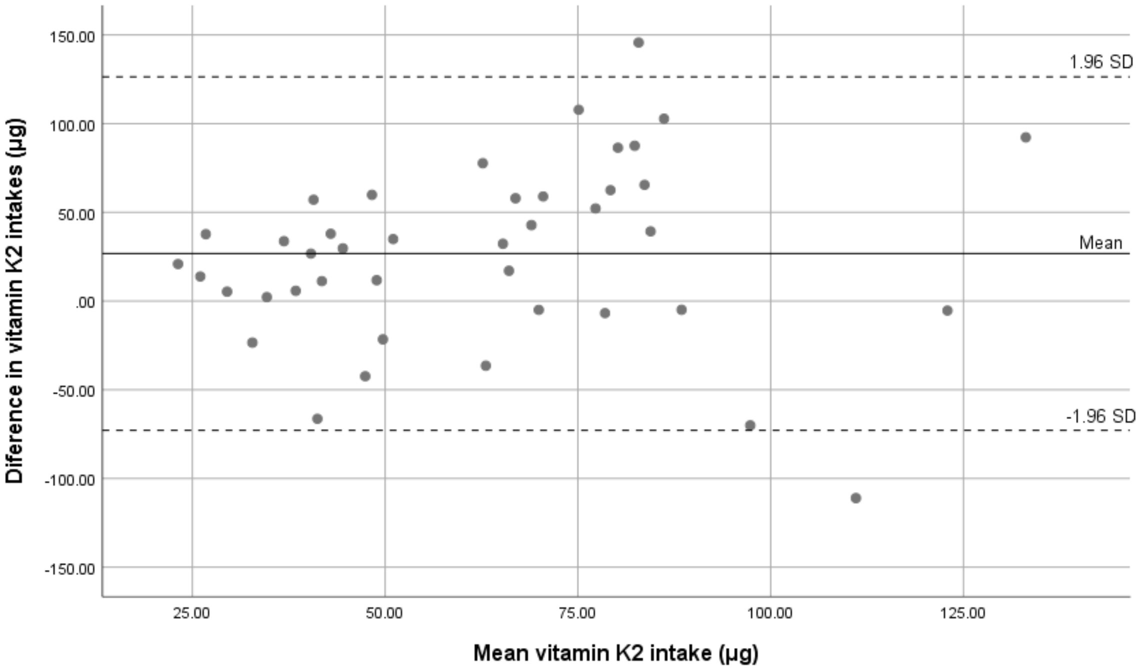

Agreement in VK2 intake between the 4-day averaged 24HDR and the final FFQ

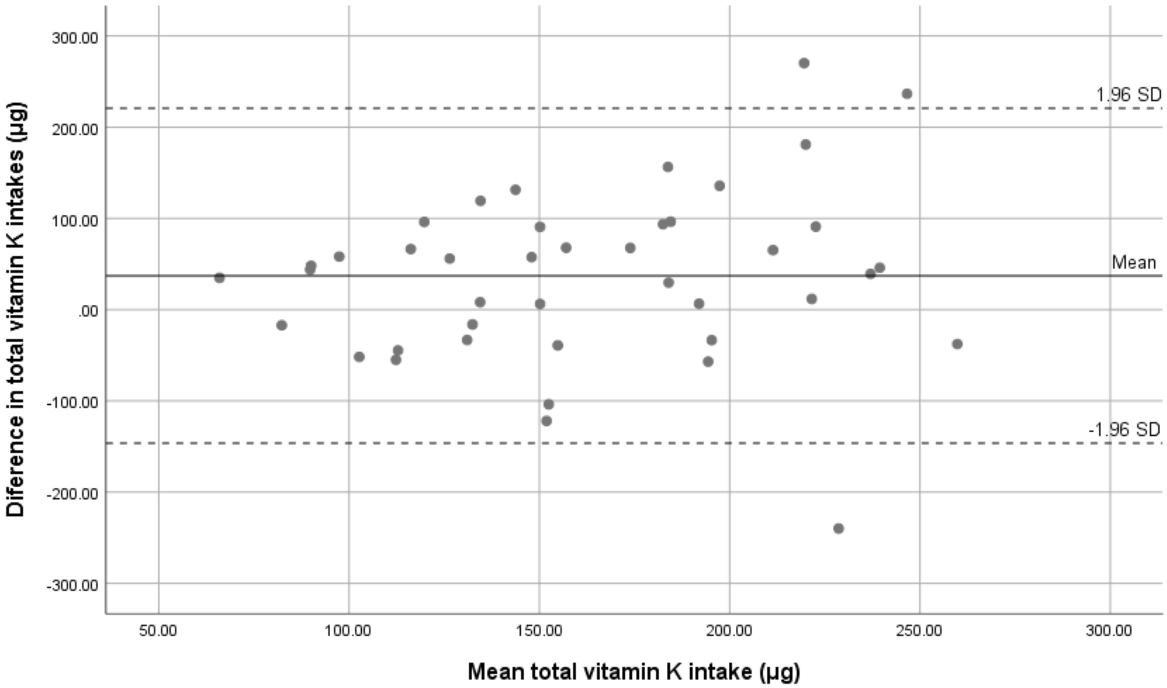

Agreement in total VK intake between the 4-day averaged 24HDR and the final FFQ
